# Posterior hemivertebra resection and short-segment fusion with lateral mass screws in congenital scoliosis: a novel strategy for the resource-limited setting

**DOI:** 10.1186/s13018-021-02419-0

**Published:** 2021-04-17

**Authors:** Mohammad Zarei, Ehsan Ghadimi, Nima Bagheri, Seyed Mir Mansour Moazen Jamshidi, Alireza Moharrami, Mersad Moosavi, Soroush Baghdadi

**Affiliations:** 1 Joint Reconstruction Research Center, Tehran University of Medical Sciences, Keshavarz Blvd, Tehran, 1419733141 Iran; 2 The Children’s Hospital of Philadelphia, Division of Orthopaedics, Philadelphia, PA USA

**Keywords:** Congenital scoliosis, Congenital deformity, Hemivertebra, Hemivertebra resection, Lateral mass screw, Posterior spinal fusion, Complication

## Abstract

**Background:**

Posterior hemivertebra resection and short-segment fusion with pedicle screws are an established treatment in congenital scoliosis, which require pediatric-specific instrumentation. The purpose of this study was to report the results of utilizing cervical lateral mass screws instead of pedicle screws in the treatment of congenital scoliosis in children younger than 5 years old.

**Methods:**

In an IRB-approved retrospective chart review study, patients <5 years old with congenital scoliosis who underwent posterior hemivertebra resection and fusion with lateral mass screws from 2013 to 2017 were included. Demographic information, pre- and post-operative radiographs, complications, and outcomes were extracted from the charts.

**Results:**

Twenty-three patients were included in the final analysis with a mean age of 40 months, of which 14 were female. Patients were followed for a mean of 51.3±13.2 months. The mean blood loss was 210ml, and patients were hospitalized for a mean of 4 days post-operatively. The correction rate of the main coronal curve, compensatory cranial curve, compensatory caudal curve, and segmental sagittal curve was 74.8%, 68%, 65.2%, and 68.9%, respectively. Three complications were observed: one intra-operative pedicle fracture, one case of implant failure, and one deep surgical-site infection, all of which were successfully managed.

**Conclusions:**

Our findings suggest that adult lateral mass screws can be used for transpedicular fixation of the thoracic and lumbar vertebrae in low-resource settings where pediatric-specific pedicle instruments are not readily available. The correction rate, outcomes, and complications are similar and comparable to pediatric-specific pedicle screws, in addition to being low-profile and less bulky compared to adult implants.

## Background

Hemivertebra is the most common form of congenital scoliosis, resulting from a failure of normal vertebral formation [1]. The treatment is challenging in many cases and largely depends on the number, location, and type of congenital hemivertebra, as well as age and concurrent pathologies. Long unfused segments are amenable to guided growth procedures. However, isolated hemivertebra have the highest potential for curve progression, and as such, are best treated with resection and short-segment posterior spinal fusion [2]. Such a strategy has been shown to provide excellent results, especially when done early (3–5 years) [3].

**Fig. 1:**
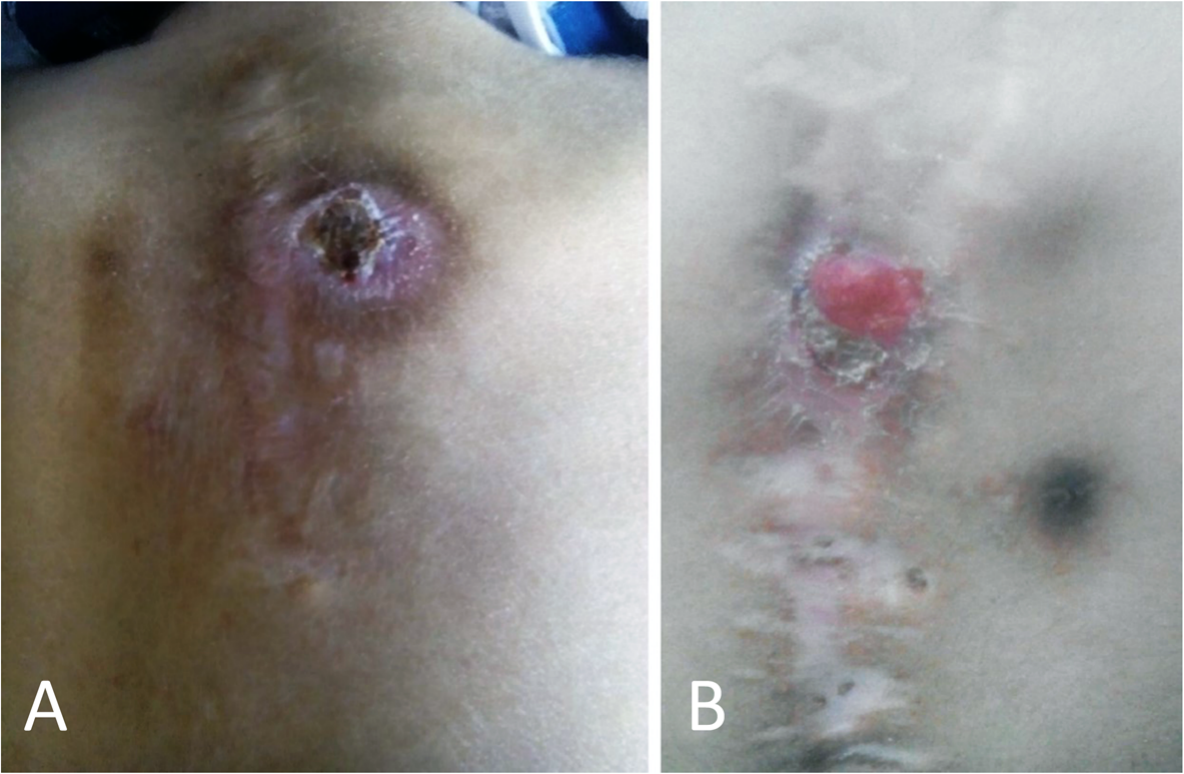
A 25-month-old boy (**a**) and 30-month-old girl (**b**) with scoliosis, operated by adult-specific pedicle screws. Note hardware prominence leading to skin breakdown. Revision surgery was done for both patients

Posterior fusion in young children requires utilizing pediatric-specific instrumentation systems, as 3.5-mm pedicle screws are often needed due to the small size of the pediatric vertebrae. Pediatric-specific instruments are not available in many centers, especially in the developing world, due to their cost and limited applications [4, 5]. An alternative is to operate when the patient is older and use adult 4.5- or 5-mm pedicle screws. However, these screws are bulky, and such hardware’s prominence might be problematic and even cause the overlying skin’s breakdown (Fig. 1). Furthermore, there is a potential risk of pedicle screw fracture when using adult-type screws, which might be challenging to manage and cause devastating complications.

**Fig. 2:**
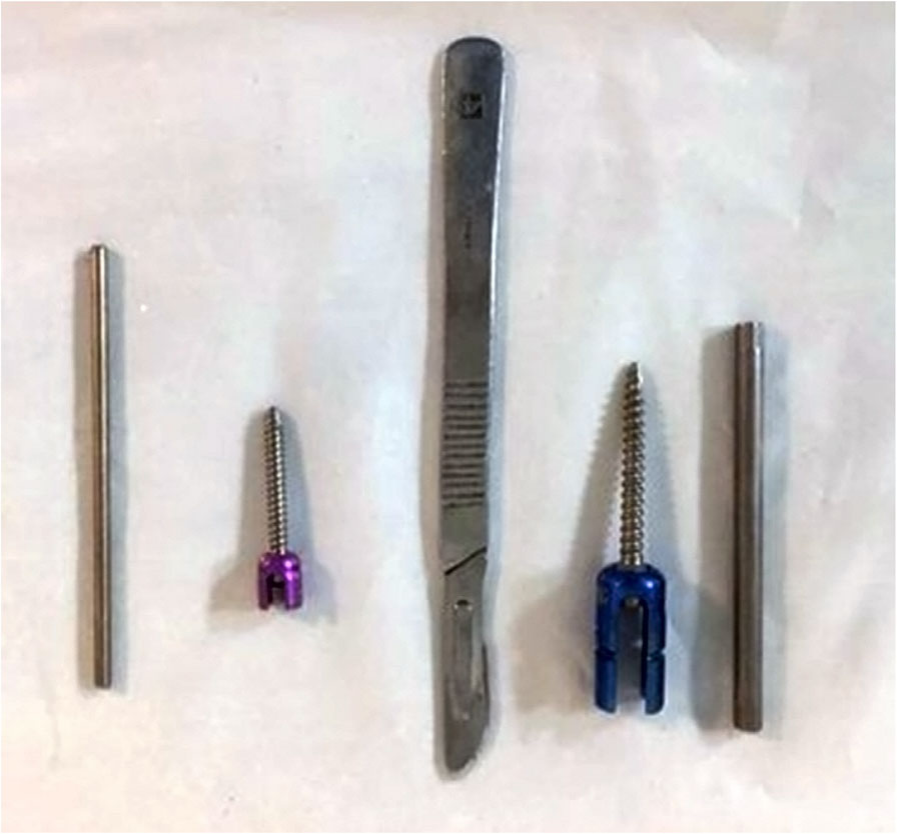
A 3.5-mm cervical lateral mass screw and rod (left) and a 4.5-mm adult-specific pedicle screw and rod (right)

At our institution in Tehran, Iran, we have had limited and intermittent access to pediatric pedicle screw instrumentation throughout the years and have been using cervical lateral mass screws and rods as pedicle screws to treat congenital scoliosis. Lateral mass screws are low-profile (Fig. 2), are more readily available, and in our experience, have been a good alternative to pediatric pedicle screws. Therefore, this study was performed to report the posterior fusion results with cervical lateral mass screws in isolated hemivertebra treatment and evaluate possible complications and safety issues.

## Methods

After obtaining IRB approval, a retrospective chart review was done to identify patients who underwent hemivertebra resection and posterior fusion at our institution between 2013 and 2017. Patients under the age of 5 years were selected because the ideal application of this technique is in this age group. Patients who underwent a posterior fusion with lateral mass screws were selected and included in the final analysis from this database. Exclusion criteria included patients diagnosed with non-congenital curves, patients older than 5 years of age, and patients who required extensive surgery, defined as more than a single-level hemivertebra resection. Patients who had undergone previous surgeries were also excluded, as well as patients with <2 years of follow-up. All surgeries were done by a fellowship-trained orthopedic spine surgeon (MZ) at a referral spine center.

Demographic data were collected, and radiographic measurements were performed on pre-operative, immediate post-operative, and follow-up standing full spine posteroanterior and lateral radiographs. The main curve, compensatory cranial curve, and compensatory caudal curve were identified and measured by the senior author. To assess sagittal plane correction, segmental kyphosis or lordosis was compared with Bernhardt and Bridwell’s corresponding standard values and was reported as positive for kyphosis and negative for lordosis [6]. Intra-operative blood loss, operative time, and complications were extracted from the operative notes. Complications, either in the early or late post-operative period, were identified and recorded.

A thorough physical examination and radiographic evaluation were performed for accompanying congenital anomalies. A pre-operative CT scan with reconstruction was done to choose the appropriate instrument and evaluate the pedicle morphology. To assess intracanal anomalies, a pre-operative spine MRI was performed in all patients, which revealed syringomyelia in one patient and a tethered cord, neither of which required a change in the surgical plan.

### Surgical technique

All patients underwent a standard resection of the hemivertebra using a single-stage posterior approach. Fixation and fusion of the adjacent vertebrae were performed using transpedicular instrumentation with lateral mass screws and rod.

**Fig. 3:**
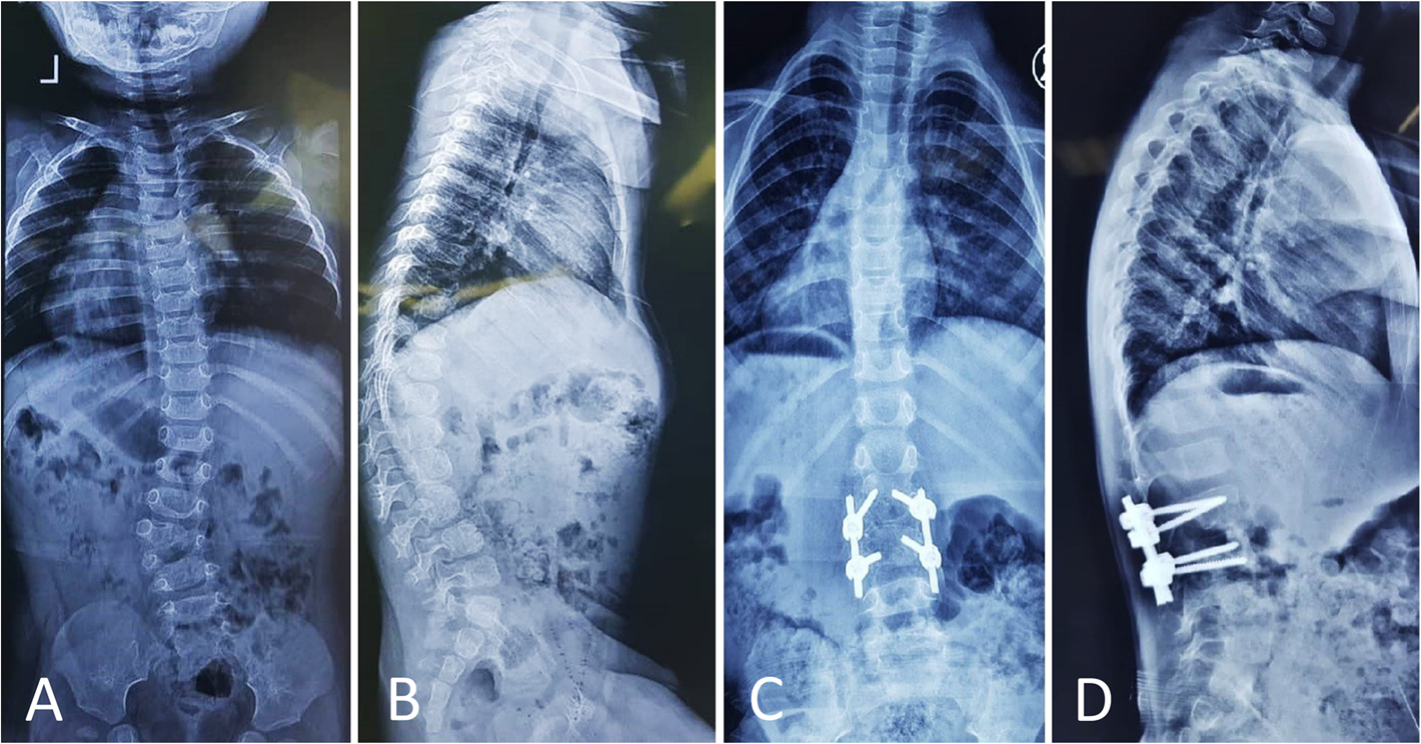
An 18-month-old boy with congenital lumbar hemivertebra. Pre-operative radiographs (**a**, **b**) show a fully segmented hemivertebra in the lumbar region with coronal segmental scoliosis of 39° and segmental kyphosis of 16°. Post-operative radiographs (**c**, **d**) after hemivertebra resection and internal fixation with lateral mass screws. The coronal curve was corrected to 5° and the sagittal to 1°

All screws were placed with a free-hand technique, and a correct placement was confirmed with fluoroscopy. The thoracic vertebrae’s starting point was the intersection of a vertical line immediately lateral to the midpoint of the facet joint and a horizontal line at the superior third of the transverse process. In the lumbar spine, the intersection of a vertical line that passes along the lateral aspect of the facet joint and a horizontal line that bisects the transverse process was chosen as the entry point. Using a rongeur, the entry point was decorticated, and a passage in the pedicle was initially gained using a 2-mm curved probe. The pedicle feeler was used to check the passage. A 3.5- or 4-mm polyaxial lateral mass screw with an appropriate length was subsequently inserted into the pedicle (Fig. 3). The fusion only included the two vertebrae adjacent to the resected hemivertebra. In case of a significant kyphosis, severe scoliosis, pedicle fracture, or insecure fixation, fusion was extended for one or two additional segments.

A standard posterior hemivertebra resection was performed with resection of the facets, lamina, and the hemivertebra’s transverse process to expose the pedicle. The upper and lower nerve roots were then explored. In the thoracic spine, the transverse process, the rib head, and the proximal part of the rib on the convex side were resected. The pedicle’s remnants, the discs adjacent to the hemivertebra, and the hemivertebra’s vertebral body were removed, and the adjacent vertebra end plates are debrided down to bleeding bone. In cases with a large hemivertebra or with substantial kyphosis, an autologous bone graft and mesh cage were used to provide stability and correct the kyphosis. After contouring and insertion of the rod, compression was applied on the convex side until the gap was closed completely. If the gap persisted, cancellous bone graft was used in the gap.

All patients were intraoperatively monitored with somatosensory-evoked potentials (SSEP) and motor-evoked potential (MEP) neuromonitoring. Patients were mobilized in the first post-operative week, and an orthosis was used for 3–6 months when awake.

### Statistical analysis

To analyze the difference between pre-operative, post-operative, and final follow-up values, paired student’s *t* tests were used. All statistical analyses were performed using IBM SPSS Statistics for Windows, Version 22.0 (Armonk, NY). The level of significance was considered as *P*<0.05.

## Results

Twenty-three patients (14 females, nine males) were included in the final analysis, with a mean age of 40 months at the time of surgery (range, 27–60 months). Patients were followed for a mean of 51.3±13.2 months post-operatively (range, 26–88 months).

The hemivertebra was located in the thoracic spine (T1–T9) in nine patients (39%), thoracolumbar region (T10–L2) in ten patients (44%), and lumbar spine (L3–L4) in four patients (17%). Sixteen hemivertebrae (70%) were fully segmented, while seven (30%) were semi-segmented. None of the hemivertebrae was incarcerated. Associated anomalies were present in seven patients (30%), including intraspinal anomalies (3 patients), brain pathologies (2 patients), cardiopulmonary (1 patient), and genitourinary system (1 patient) anomalies.

Overall, lateral mass screws were inserted in T3 to L5 vertebrae, with a diameter of 3.5mm in the T1–T12 vertebrae and 4mm in L1–L5. The mean screw length was 26mm (range, 20–30mm) in the thoracic spine (T1–T12) and 30mm (range, 24–36mm) in the lumbar spine (L1–L5). In three patients with significant kyphosis and curves >50°, one additional segment was added to the fusion to avoid overloading the construct and reduce the risk of pedicle fracture or implant failure.

The mean operation time was 143 min (range, 90–270 min). The average blood loss was 210ml (range, 70–430ml). Patients were admitted for a median of 4 days (range, 3–7 days). All patients wore a brace for a mean of 4 months (range, 3–6.5 months) and were mobilized with a brace within the first post-operative week.

The mean Cobb angle of the main coronal curve was 42.5° before surgery (range, 18°–72°), which was corrected to a mean of 8.5° (range, 3°–39°) immediately after surgery. The mean segmental sagittal kyphosis (difference from the normal value [6]) was 19.3° preoperatively (range, 4°–36°), which was corrected to a mean of 8.8° post-operatively. While all measurements showed a significant improvement in the post-operative radiographs, the difference between immediate post-operative and latest follow-up measurements was not statistically significant (Table 1).

**Table 1:** Correction are achieved among patients in this series. Values are presented as mean±SD (range). Significant *P* values are indicated in italic

	Pre-operative	Post-operative	P value (pre-op to post-op)	Correction rate (%)	Latest follow-up	*P* value (post-op to latest follow-up)
Main curve (°)	42.5 ± 10.1 (18–72)	8.5 ± 7.2 (3–39)	*0.001*	75	10.7 ± 7.5 (2–35)	0.1
Compensatory cranial curve (°)	16.2 ± 10.7 (3–35)	4.1 ± 2.9 (1–16)	*<0.001*	68	5.1 ± 4.4 (2–16)	0.06
Compensatory caudal curve (°)	19.3 ± 12.4 (5–75)	6.2 ± 6.1 (5–34)	*<0.001*	65	6.7 ± 6.4 (3–35)	0.5
Segmental kyphosis (difference from normative values)	19.l ± 8.6 (4–36)	8.8 ± 5.1 (−2–19)	*0.002*	69	6 ± 7.1 (−4–23)	0.08

There were no neurologic, vascular, or visceral complications, respiratory problems, or death in any of the patients. The only intra-operative complication was a convex pedicle fracture in one patient, which was managed by extending the fusion by one level. None of the patients had hardware-related skin breakdown or complained of hardware prominence. One patient experienced a surgical site infection, which resolved with surgical irrigation and debridement and antibiotics. In one case, implant failure was observed due to screw pull out 1 year after the index procedure, which was treated with an uneventful revision. One patient had a 10° increase in the cobb angle of the main curve in the follow-up period, which was managed by continuing the brace for four additional months, after which the curve stabilized and went on to fuse uneventfully. Proximal junctional kyphosis was not observed in any patient.

## Discussion

In this study, we reported hemivertebra resection and posterior fusion results with lateral mass screws in 23 patients with congenital scoliosis. Our findings suggest that in the lack of pediatric-specific pedicle screws, lateral mass screws from an adult cervical set can successfully be used for posterior fusion in children under 5 years.

Pediatric-specific spinal instruments are not readily available at many centers, especially in the developing world, due to the cost and limited application of such instruments. However, adult instruments have been in the market for decades, are less expensive, and are available at any spine surgery center. The potential solutions to the lack of pediatric-specific implants are to use adult-type implants in older children, postpone surgery in younger children, and subsequently use adult implants, or use hooks instead of pedicle screws. We argue that neither of these solutions is ideal, and they might result in suboptimal outcomes and complications [7].

A swift curve progression is typically observed in segmented hemivertebrae due to their imbalanced growth potential. The curve will grow and become more rigid and hard to correct if left untreated. Also, the development of compensatory curves will further complicate treatment [8]. Early treatment diminishes the need for extensive procedures and mitigates the need to correct compensatory curves. Early resection of the hemivertebra and short-segment posterior fusion is the current standard of care in congenital hemivertebra. In a cohort of congenital scoliosis patients, Chang et al. have shown that patients treated before the age of 6 years have a significantly better deformity correction compared to after 6 years of age. They did not observe a disturbing vertebral/spinal growth in patients who received early treatment [9].

Compared with non-rigid constructs, pedicle screws offer a three-column control of the spine, a superior control to correct the deformity, higher fusion rates, and lower implant failures and pseudarthrosis [10–13]. Even in children younger than 5 years old, pedicle screw constructs are safe and efficient [14, 15]. Ruf et al. have reported the safety and efficacy of pedicle screws even in 1- and 2-year-old children [16]. We have previously used adult-type pedicle screws in children who had a sufficient pedicle diameter to accommodate larger screws but have experienced unacceptably high skin breakdown rates and complaints of prominent hardware from almost all patients/parents. We have therefore abandoned the practice despite otherwise excellent results. We have been using lateral mass screws as pedicle screws in children younger than 5 years old and have been satisfied with the technique, results, and the low rate of complications. Lateral mass screws are low-profile, readily available at most centers, and have an appropriate structure and diameter to be used as pedicle screws in children. In our series, the mean age at the surgery time was 40 months, with all patients undergoing surgery before 5 years, which is the ideal age of treatment in congenital scoliosis. The use of lateral mass screws in lieu of pediatric-specific pedicle screws has allowed us to provide the highest standard-of-care for our patients without sacrificing the results or subjecting them to undue risk of complications. While lateral mass screws are successfully used in the adult and pediatric cervical spine to provide three-column fixation, their use as pedicle screws has not been reported previously. This technique may also be combined with 3D-printed spine models or guides, which would serve as an educational tool for the family and the medical team, and also increase the precision of the pre-operative planning [17, 18].

In our study, the mean percent correction of the main coronal curve, segmental sagittal angles, compensatory caudal, and compensatory cranial curves were 74.8%, 68.9%, 68%, and 65.2%, respectively. In a retrospective study of 28 patients with congenital scoliosis, Ruf et al. [14] reported a correction rate of 72% for the main curve, 78% for the compensatory cranial curve, 65% compensatory caudal curve, and 63% for kyphosis. Other studies have also reported similar correction rates (Table 2) [3, 19, 20].

**Table 2:** Summary of previous studies

First author	Year	Cases	Mean correction rate	Implant failure	Total number of complications (%)	Repeat surgery
Ruf and Harms [14]	2003	28	69.5%	3	6 (21%)	4
Guo et al. [3]	2016	39	74.5	0	3 (7%)	4
Erden et al. [19]	2017	9	81%	0	0 (0%)	0
Xue and Zao [20]	2018	43	69%	0	4 (9.3)	4

Overall, we had three complications among our patients (13%), including one intra-operative pedicle fracture, one deep infection, and one implant failure. All complications were managed according to accepted guidelines, and neither resulted in long-term complication or morbidity. Guo et al. reported a 7% rate of complications [3], while Ruf and Harms reported a 21% rate of complications in their respective series [14]. While revision surgery for screw malposition has been reported in 0.8 to 4.3% of children and adults undergoing posterior instrumentation, none of our patients needed such a revision surgery [21–25].

This study has several limitations, including those inherent in a retrospective case series. While the small study population is in line with previous studies and is due to the procedure being relatively uncommon, it precludes making strong suggestions and recommendations. However, one surgeon performed all surgeries, and we will also be conducting follow-up studies with control groups. Additionally, we do not perform routine intra- or post-operative CT scans and, therefore, could not evaluate minor malpositions. However, none of our patients had a grossly malpositioned screw or a complication of such malposition. Finally, we did not report patient-reported outcomes in this study, although one would assume that hardware choice will not significantly affect the quality of life.

## Conclusions

Our findings suggest that adult lateral mass screws can be used for transpedicular fixation of the thoracic and lumbar vertebrae in low-resource settings where pediatric-specific pedicle instruments are not readily available. The correction rate, outcomes, and complications are similar and comparable to pediatric-specific pedicle screws, in addition to being low-profile and less bulky compared to adult implants.

## Data Availability

The datasets used and/or analyzed during the current study are available from the corresponding author on reasonable request.

## Abbreviations

CT: Computed tomography
MRI: Magnetic resonance imaging
SSEP: Somatosensory evoked potentials
MEP: Motor-evoked potentials
PJK: Proximal junctional kyphosis
